# Fully Covered Self-Expandable Metal Stents for Treatment of Post-Sphincterotomy Bleeding

**DOI:** 10.4021/gr435w

**Published:** 2012-07-20

**Authors:** Ahmed Abdel Samie, Lorenz Theilmann

**Affiliations:** a Department of Gastroenterology, Pforzheim Hospital, Germany

**Keywords:** Completely covered self-expandable metal stents, Endoscopic sphincterotomy, Post-sphincterotomy bleeding

## Abstract

Endoscopic biliary sphincterotomy (ES) is the cornerstone of therapeutic endoscopic retrograde cholangiopancreatography (ERCP); however, serious complications are not uncommon. Post-sphincterotomy bleeding is one of the most frequent complications following ES and may occur in up to 10% of the patients. The spectrum of presentation may range from self-limited to severe live threatening hemorrhage. Different endoscopic treatment options are available. Angiographic embolisation and surgery are preserved for refractory cases not controlled by endoscopic means. Recently, completely covered self-expandable metal stents (CSEMS) have been applied to achieve hemostasis in severe post-sphincterotomy bleeding not controlled by other measures. We present our experience with this method to control delayed bleeding after ES in two patients requiring continuous therapeutic anticoagulation due to high cardiovascular embolic risk.

## Introduction

Bleeding, which is in the majority of cases self-limited, is one of the most common complications of endoscopic sphincterotomy. Nevertheless, severe hemorrhage can occur and may be difficult to manage. Lately, completely covered self-expandable metal stents (CESMS) have been applied for different benign biliary indications, including severe/refractory post-sphincterotomy bleeding. However, data are scarce.

## Case Report

### Case 1

A 70-year-old female patient was admitted to our unit because of abdominal pain and elevated liver enzymes (Bilirubin 2.4 mg/dL, AST 70 U/L, ALT 120 U/L, GGT 700 U/L, AP 400 U/L). The patient was on phenprocoumon because of pulmonary artery embolism four weeks previously. Abdominal sonography showed gallstones with a prominent common bile duct.

Because of a high suspicion index for Choledocholithiasis depending on the clinical, laboratory, and sonographic findings we moved next to ERCP. Phenprocoumon was stopped (INR at the time of the procedure 1.0) and the patient underwent therapeutic ERCP with ES and basket stone extraction using a standard duodenoscope and standard sphinctertom-based technique on a guide wire. Therapeutic anticoagulation was resumed with weight adapted low molecular weight heparin six hours following the procedure. Tow days later the patient presented with melena and homodynamic instability. Laboratory tests showed a hemoglobin drop to 8 g/dL (initially 12 g/dL). Endoscopy was carried out after resuscitation and transfusion of two units of packed red bloods cells.

On Endoscopy oozing from the sphincterotomy site was detected. Hemostasis was achieved via injection of 4 mL of dilute epinephrine (1:10,000).

**Figure 1:**
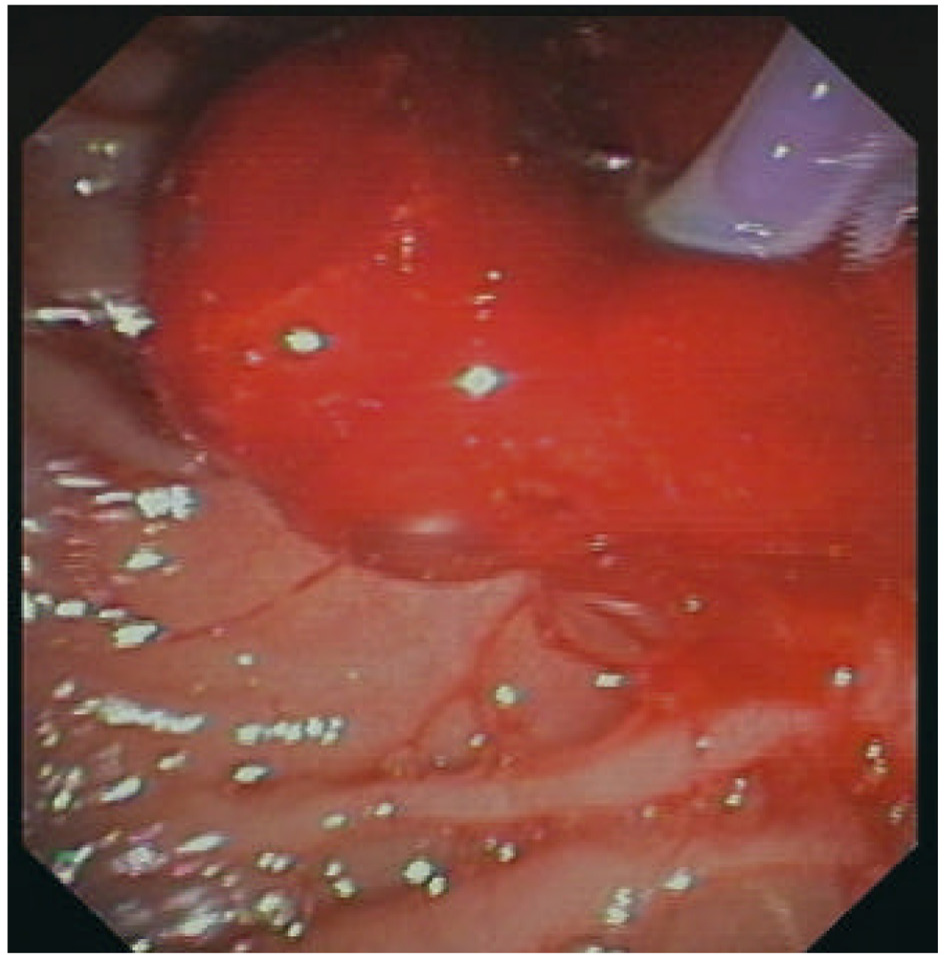
Delayed bleeding with oozing and blood clot.

**Figure 2:**
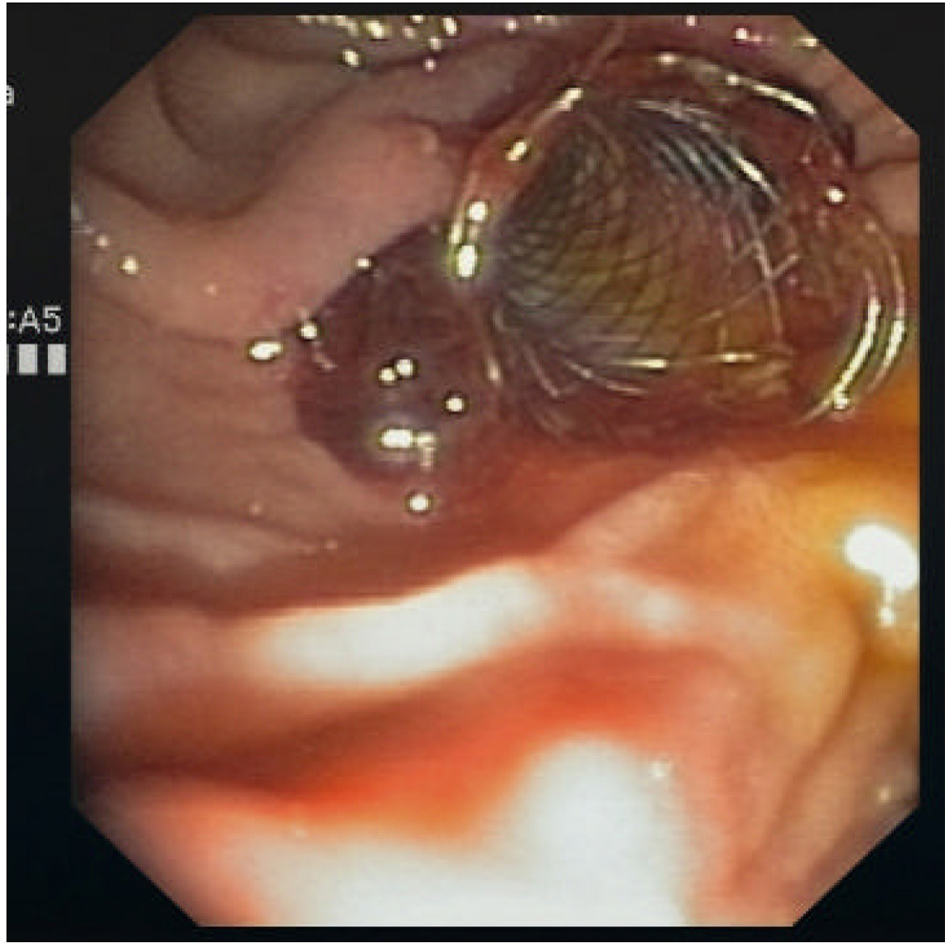
CSEMS across the papilla.

However, clinically manifest re-bleeding occurred 24 hours later (Fig. 1), requiring aggressive volume replacement and transfusion of another three units of packed red blood cells to achieve homodynamic stability. As endoscopic injection failed to control bleeding, we decided to tampon the hemorrhagic site using CSEMS. Bleeding was successfully managed with the placement of a fully covered self-expandable metal stent (WallFlex RX, Boston Scientific, 10 mm in diameter and 4 cm long) across the papilla compressing the bleeding site and achieving mechanical hemostasis (Fig. 2). Because of the high cardiovascular embolic risk of our patient, therapeutic anticoagulation was continued with LMWH on the next day and phenprocoumon was started after three days. There was no clinical evidence of re-bleeding and the stent was endoscopically removed eight days later with an alligator-tooth forceps.

### Case 2

A 76-year-old female patient was admitted to the hospital with epigastric pain. The patient was on permanent phenprocoumon therapy after mechanical mitral valve replacement. Past medical history included in addition coronary heart disease with previous myocardial infarction and coronary bypass surgery. Cholecystectomy was preformed ten years before due to symptomatic Cholecystolithiasis. Laboratory findings revealed cholestasis (Bilirubin 2 mg/dL, AST 65 U/L, ALT 75 U/L, GGT 580 U/L, AP 440 U/L). On sonography, the bile duct was 11 mm in diameter, however the distal bile duct could not be visualized because of meteorismus. Due to high index of suspicion of choledocholithiasis ERCP was indicated.

**Figure 3:**
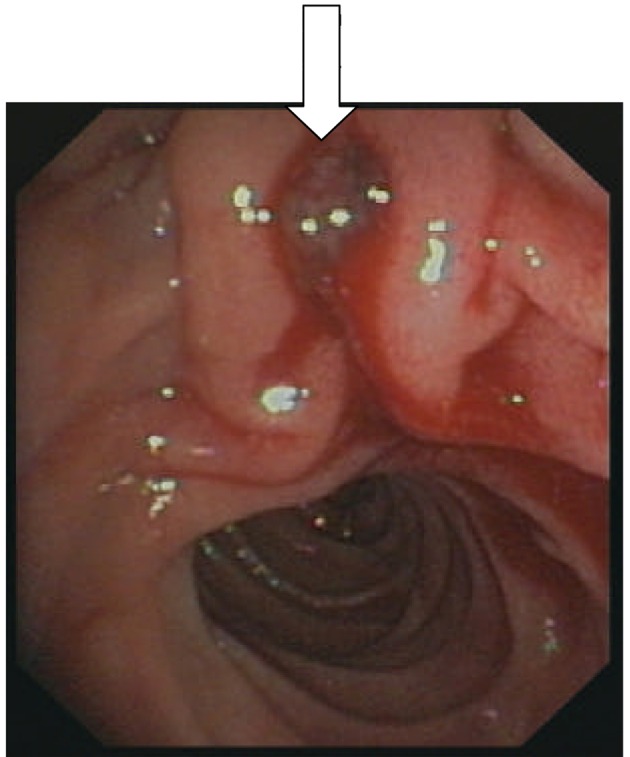
Bleeding vessel (arrow).

Phenprocoumon was stooped and bridging was started with weight adapted therapeutic dose of low molecular weight heparin (LMWH). ERCP was performed with smooth cannulation of the bile duct, ES in standard technique on a guide wire, and sludge extraction with a balloon catheter/dormia basket with no immediate complications. Therapeutic anticoagulation was started again six hours following the procedure, but the patient presented with melena and hemoglobin drop on the following day (hemoglobin 9 g/dL, initially 13 g/dL). After resuscitation, endoscopy was performed showing a bleeding visible vessel (Fig. 3) and endoscopic hemostasis was achieved with injection therapy (3 mL dilute epinephrine 1:10,000).

**Figure 4:**
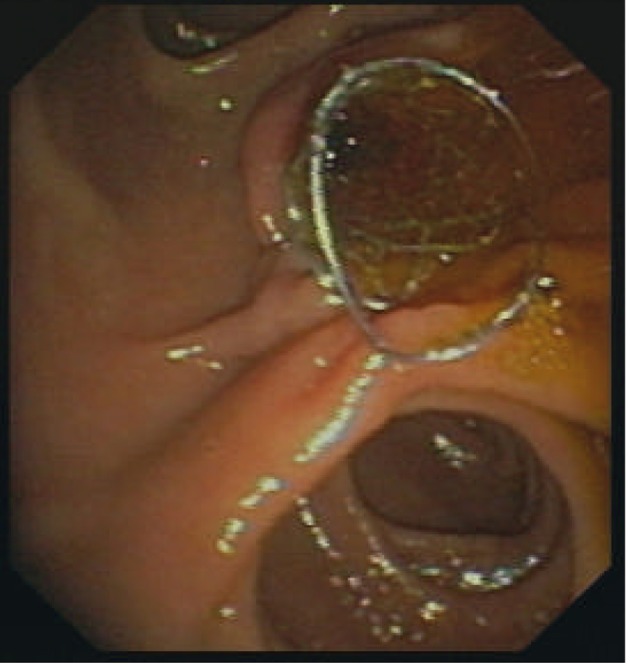
CSEMS compressing the bleeding site.

Due to clinically significant recurrent bleeding repeated endoscopy was indicated. Again, injection therapy with dilute epinephrine was unsuccessful to control hemorrhage, nevertheless, hemostasis was achieved by placing a CESMS across the papilla, 10 mm in diameter and 4 cm long (Fig. 4). Due to mechanical mitral valve replacement with high cardiovascular embolic risk, therapeutic anticoagulation with LMWH was continued without interruption, and phenprocoumon was started again without clinical evidence of re-bleeding. The stent was endoscopically removed two weeks later on an ambulatory basis.

## Discussion

Bleeding is one of the most common complications following endoscopic sphincterotomy.

The incidence of post-sphincterotomy bleeding reported in the literature widely varies because of differences in definition and may reach up to 10 % [1].

Established risk factors of bleeding include uncorrected coagulopathy at the time of endoscopy, use of anticoagulants within three days prior to the procedure, and acute cholangitis. In addition, the presence of a periampullary diverticulum, the use of precut technique, and low endoscopist experience may increase the risk of bleeding [2]. Our two patients were on permanent phenprocoumon therapy, which was stopped prior to the endoscopic procedure, but bridging with low molecular weight heparin was necessary, because of the high cardiovascular embolic risk in both patients. This may have contributed to the clinically relevant delayed bleeding, which occurred in both cases.

Post-sphincterotomy bleeding may vary from a mild self-limited condition to a severe life-threatening bleeding, which can be difficult to mange. Up to 2 % of the patients undergoing ES may experience massive bleeding, which often results from an injured aberrant retroduodenal artery [3].

Post-sphincterotomy bleeding can be immediate occurring at the time of the procedure or delayed. Delayed bleeding occurs from hours up to ten days after ES. Immediate bleeding is more frequent than delayed bleeding. The bleeding can be non clinically significant or clinically significant, which is further classified as mild, moderate and severe, depending on clinical presentation, hemodynamic parameters and fall in hemoglobin level [2].

As most of the bleeding episodes stops spontaneously, endoscopic therapy is preserved for endoscopically significant immediate bleeding and clinically relevant delayed bleeding.

Endoscopic therapy includes injection, thermal, and mechanical therapy using balloon tamponade or endoclips. Injection of diluted epinephrine (1:10,000) in and around the sphincterotomy site is the most commonly used method, whereas the amount of injected solution may vary from 0.5 to 30 mL [3].

Nevertheless, application of endoscopic therapy may be technically very demanding due to failure of exact localization of the bleeding site during severe hemorrhage and difficulty in maneuvering instruments through a side-view endoscope. Furthermore, the risk of pancreatitis may increase if endoscopic combination therapy is applied [4].

Angiographic Embolisation and surgery are preserved for refractory bleeding not responding to endoscopic measures.

Fully covered self-expandable metal stents (CSEMS) intended to palliate malignant biliary conditions, were recently used for different non malignant biliary indications, including benign biliary strictures, post-operative bile duct leakage, periampullary perforation due to endoscopic sphincterotomy (type 2), and post-sphincterotomy bleeding [5].

In a recent retrospective analysis including 11 patients, hemostasis was achieved in all patients using CSEMS after failure of other measures. The mean duration of stent placement was 8.2 days and all stents were successfully removed endoscopically [6]. In another case series, including five patients CESMS were effective to control bleeding in all patients. The stents were removed within eight weeks in three patients and migrated spontaneously without clinical sequelae in two patients [7].

In a multicenter study (37 patients) removal attempts of CSEMS were successful in all cases [8]. The endoscopic feasibility and safety of stent removal were also documented by other authors [9].

A further advantage of this treatment modality is the simultaneous and effective drainage of the bile duct, especially if occluded with blood clots. Stent dislocation, however, represent a drawback of this device, particularly if used in treating benign biliary disorders.

Injection therapy failed to control bleeding in our two patients. Temporarily placement of CSEMS achieved hemostasis in both patients and the stents were easily removed endoscopically eight and fourteen days after placement respectively. Furthermore, because of the high cardiovascular embolic risk in both patients therapeutic anticoagulation was continued with LMWH after stent placement and oral anticoagulation with phenprocoumon could be safely resumed in both cases.

### Conclusion

Temporarily placement of fully covered self-expandable metal stents may represent an effective mechanical measure to achieve endoscopic hemostasis in post-sphincterotomy bleeding not controlled by other means, however data are scarce.

To our Knowledge, this is the first case report documenting the effectiveness of this measure in refractory bleeding following endoscopic sphincterotomy in patients on therapeutic anticoagulation because of high cardiovascular embolic risk.
